# WNT enhancing signals in pancreatic cancer are transmitted by LGR6

**DOI:** 10.18632/aging.205101

**Published:** 2023-09-27

**Authors:** Jing Wang, Dominik T. Koch, Felix O. Hofmann, Daniel Härtwig, Iris Beirith, Klaus Peter Janssen, Alexandr V. Bazhin, Hanno Niess, Jens Werner, Bernhard W. Renz, Matthias Ilmer

**Affiliations:** 1 Department of General, Visceral and Transplantation Surgery, Hospital of the University of Munich, Ludwig-Maximilians-University (LMU), Munich, Bavaria, Germany; 2 Department of General Surgery, The First Affiliated Hospital of USTC, Division of Life Sciences and Medicine, University of Science and Technology of China, Hefei, Anhui, P.R. China; 3 Department of Surgery, School of Medicine, Klinikum Rechts der Isar, Technical University of Munich, Munich, Bavaria, Germany; 4 German Cancer Consortium (DKTK), Partner Site Munich, Munich, Bavaria, Germany; 5 Bavarian Cancer Research Center (BZKF), LMU Munich, Munich, Bavaria, Germany

**Keywords:** pancreatic ductal adenocarcinoma, WNT signaling, epithelial-mesenchymal transition, LGR6, cancer stem cells

## Abstract

The G-protein-coupled receptor LGR6 associates with ligands of the R-Spondin (RSPO) family to potentiate preexisting signals of the canonical WNT pathway. However, its importance in pancreatic ductal adenocarcinoma (PDAC) remains unclear. Here, we show that LGR6 is differentially expressed in various PDAC cell lines of mesenchymal and epithelial phenotype, respectively, siding with the latter subsets. LGR6 expression is altered based upon the cells’ WNT activation status. Furthermore, extrinsic enhancement of WNT pathway signaling increased LGR6 expression suggestive of a reinforcing self-regulatory loop in highly WNT susceptible cells. Downregulation of LGR6 on the other hand, seemed to tamper those effects. Last, downregulation of LGR6 reduced cancer stemness as determined by functional *in vitro* assays. These findings shed new insights into regulatory mechanisms for the canonical WNT pathway in pancreatic cancer cells. It may also have potential value for treatment stratification of PDAC.

## INTRODUCTION

Pancreatic ductal adenocarcinoma (PDAC) is one of the most aggressive malignancies with an overall 5-year survival rate of 10% for all stages combined [1, 2]. By 2030, current predictions see the disease as the second most common cause of cancer-related death [3]. Regulation of PDAC on a cellular level during early and later pancreatic tumorigenesis remains incompletely understood and hence, presents challenges for better treatment options. Precise regulation of the canonical WNT signaling pathway is fundamental for normal development on the one hand as well as tissue regeneration of various origins on the other hand [4, 5]. WNT signaling has also been shown to conduct critical functions in physiologic pancreatic development [6, 7]. Although aberrant WNT signaling has been linked to tumorigenesis in multiple organs [8–11], typical activating mutations, such as in APC or β-catenin, are not commonly seen in PDAC cells with the exception of RNF43 [12, 13]. However, it was shown in some pancreatic tumors that an elevated nuclear accumulation of β-catenin indicative of activated WNT signaling correlated with progression of PDAC [14, 15].

The WNT pathway gets activated upon the engagement of canonical WNT ligands to their co-receptors low-density lipoprotein receptor-related protein (LRP) 5 or 6 and frizzled. Intracellularly, the transcription factor β-catenin escapes from its destruction complex in the cytosol comprised of APC, AXIN2, and GSK3β. Protein kinase A (PKA) phosphorylates β-catenin at Ser^675^ and thereby induces its subsequent accumulation and translocation into the nucleus, where it associates with TCF/LEF binding sites (T-cell factor / lymphoid enhancer factor) regulating canonical WNT target gene expression [16, 17]. The R-spondin (RSPO) family is a group of secreted factors which enhance a previously activated WNT signal by binding to Leucine-rich repeat containing G-protein coupled Receptors 4/5/6 (LGR4/5/6). This mechanism normally implicates the interaction of the LGR/RSPO-complex with transmembrane E3 ubiquitin ligases ZNRF3/RNF43 [18]. These ligases execute negative feedback on canonical WNT activity by promoting the ubiquitination and hence, the inactivation of the WNT co-receptors frizzled and LRP6 [18, 19]. Upon the association of RSPO with LGR, ZNRF3/RNF43 is removed from the cell surface and subsequently, the brakes on WNT signaling are released [18].

One process by which epithelial cells disconnect from each other and transdifferentiate into mesenchymal cells is the so-called epithelial–mesenchymal transition (EMT) [20–22]. EMT and canonical WNT signaling are intertwined through the key mediator β-catenin, which is also part of the cell-cell adhesion complex that consists of different catenin proteins (α, β) and E-cadherin [23, 24]. In this regard, E-cadherin acts as a negative regulator of WNT signaling through its recruitment of β-catenin to adherence junctions. Upon induction of EMT, loss of E-cadherin leads to release of β-catenin and its accumulation in the nucleus, which subsequently activates WNT signaling [25–27]. Meanwhile, high WNT activity has been shown to activate the EMT transcription factors SNAIL2 directly or ZEB1 indirectly to induce EMT, suggesting a feedforward loop of when cancer cells undergo processes of dedifferentiation [28].

LGR5 has been serially described as a WNT target gene as well as a marker for (cancer) stem cells in multiple neoplasms [29–31]. Whether its homologue LGR6 incorporates similar functional aspects, still remains to be answered. In this work, we aimed to decipher the functions of LGR6 in WNT signaling of PDAC, apart from its assumed assignment as a receptor to RSPO. Taken into account the connections between WNT signaling and EMT, we further hypothesized a likely interplay of LGR6 and EMT.

## MATERIALS AND METHODS

### Cell culture

Human pancreatic cancer cell lines (BxPC3, Capan2, MiaPaCa2, Panc1) were obtained from American Type Culture Collection (ATCC). Cells were cultured in RPMI1640 (Lonza, Basel, Switzerland). Media were supplemented with 10% Fetal Bovine Serum (FBS; Biochrom, Cambridge, UK) and 1% penicillin/streptomycin (Lonza). All cell cultures were kept at 5% CO_2_ at 37° C. Following our internal Standard Operating Procedures (SOP), routine cell testing for mycoplasma was performed every four months. Cell authentication was conducted by IDEXX BioResearch once a year (Ludwigsburg, Germany).

### Reagents

Recombinant human proteins WNT3a and RSPO2 were purchased from R&D systems (Wiesbaden, Germany) and PeproTech® (Hamburg, Germany), respectively. TGFβ1 and U0126 were from ImmunoTools (Friesoythe, Germany) and Cell Signaling Technology® (Frankfurt am Main, Germany), respectively. IWP2 was purchased from Selleck Chemicals (Munich, Germany).

### GSEA

Gene set enrichment analysis (GSEA) of public datasets was performed with the GSEApreRanked tool of the GSEA 4.1.0 Desktop Application and following the guidelines published by the Broad Institute (http://www.broadinstitute.org/gsea/index.jsp). Hallmark gene sets were collected from the Molecular Signature Database (MSigDB). Data derived from pancreatic cancer patients were selected from the TCGA database (Project ID: TCGA-PAAD) and normalized enrichment scores (NES) were calculated according to the ranked-ordered gene list. When NES>1 or <-1, it was considered as “enriched”, showing a positive or negative correlation of selected gene sets in PAAD LGR6corr patients.

### Sphere-formation assay (SFA)

Adherent cells were trypsinized and subsequently resuspended as single cell suspensions. 1,000 cells were seeded in 100μl/well into low attachment 96-well plates (Corning, Krailling, Germany) in CSC medium. The FBS-depleted CSC medium was supplemented with 1XB27 (Thermo Fisher Scientific GmbH, Dreieich, Germany), 1% penicillin/streptomycin, recombinant human epidermal growth factor 10ng/ml and recombinant human fibroblast growth factor 20ng/ml (ImmunoTools, Friesoythe, Germany) in DMEM/F12 (Thermo Fisher Scientific GmbH, Dreieich, Germany). To prevent cell-cell aggregation, 1% methylcellulose (Sigma-Aldrich Chemie GmbH, Munich, Germany) was added to the medium. Medium changes occurred every 3-4 days. Spheres were counted after an incubation period of 12-15 days at 37° C.

### Colony-formation assay (CFA)

After thorough counting, 1,000 cells were seeded into 6-well plates in regular complete culture medium as described above. The colonies were incubated at 37° C with 5% CO2 for 7-10 days. Colonies were then fixed with 4% PFA and stained with crystal violet (Sigma GmbH, Rödermark, Germany). Cell clusters with approximately 50 cells or more were considered and counted as a colony.

### siRNA transfection

For siRNA transfection, PDAC cells were seeded at 60% confluence into 24-well plates in RPMI1640 with 10% FBS only. After overnight incubation, the old medium was removed and new RPMI1640 without penicillin/streptomycin and FBS was added. According to the supplier’s instructions, LGR6 siPOOL (siTOOLs Biotech, Martinsried, Germany) and Lipofectamine RNAiMAX Transfection Reagent (Thermo Fisher Scientific) were mixed at a ratio of 1:1 and added into the medium. The control group was prepared with negative control (nonsense) siRNA and Lipofectamine RNAiMAX Transfection Reagent. RNA was then isolated 48h after transfection. For SFA and CFA, cells were seeded 24h after transfection as described above.

### Super TOP/FOP assay

Transient transfections for SuperTOP/FOP vector assays were carried out as detailed previously [32]. Normalization was carried out with Renilla luciferase vector pRLTK (Promega, Madison, WA, USA). Passive lysis buffer was added and firefly / Renilla luciferase activity was determined with the Dual Luciferase Reporter Assay System (Promega) in triplicates.

### RNA extraction, reverse transcription, and qPCR

Total RNA from cultured cells was extracted using the total RNA isolation kit peqGOLD (VWR™/Avantor™) according to the manufacturer’s instructions. Concentration and purity of extracted RNAs were verified by a NanoDrop™ 2000 spectrophotometer (Thermo Fisher Scientific). cDNA transcription was accomplished using the cDNA synthesis kit (Bio-Rad Laboratories GmbH, Feldkirchen, Germany). qPCR was performed with the Sso Fast™ EvaGreen® kit (Bio-Rad); for normalization of gene of interest expression, we used the housekeeping gene GAPDH.

### Western blot

Treated cells were lysed in RIPA buffer including complete protease and phosphatase inhibitor cocktails (Roche). Gel electrophoresis of cell lysates was carried out on 12% SDS-PAGE gels and later transferred onto PVDF membranes. Membranes were incubated with primary antibodies overnight at 4° C and HPR-conjugated secondary antibodies for 1h at room temperature. Exposure was performed using ECL™ Western Blotting Detection Reagent (GE Healthcare, Munich, Germany). All antibodies are listed in Supplementary Table 1.

### Immunofluorescence staining

Cells were seeded onto coverslips, fixed with 4% PFA for 20 mins, and followed by blocking steps with endogenous peroxidase and permeabilization with 0.1% Triton X-100/PBS. After blocking with 5% goat serum, the slides were incubated with primary antibodies at 4° C overnight, followed by secondary antibody incubation at room temperature for 1h in the dark. Slides were counterstained with DAPI (labeling and detection) and then covered with Fluorescence Mounting Medium (VECTASHIELD® Antifade Mounting Medium H-1000, Vector Labs, Eching, Germany). All primary antibodies used here were itemized in Supplementary Table 1.

### Flow cytometry

After culturing in regular medium for 24h, cells were trypsinized, washed, and collected. For analysis of membrane-bound LGR6, cells were incubated with human LGR6 APC-conjugated antibody (10μL/10^6^cells) (R&D Systems ®, FAB8458A-025) for 30 mins at RT. In contrast, cytoplasmic LGR6 detection included fixation of the cells with IC fixation buffer and washing steps with permeabilization buffer (both from eBioscience, Waltham, MA, USA). Then, cells were incubated with anti-LGR6 antibody solution for 30 mins at RT. After washing, cells were analyzed by a BD LSRFortessa™ cell analyzer (BD Biosciences, Franklin lakes, NJ, USA). Negative controls were prepared likewise.

### Statistical analysis

Statistical analysis was performed by GraphPad Prism 9 (GraphPad Software, San Diego, USA). Data are represented as mean ± SEM. Significance levels were calculated by t-test. P<0.05 was considered as statistically significant.

## RESULTS

### LGR6 expression correlates with epithelial phenotype in PDAC cell lines

**Figure 1:**
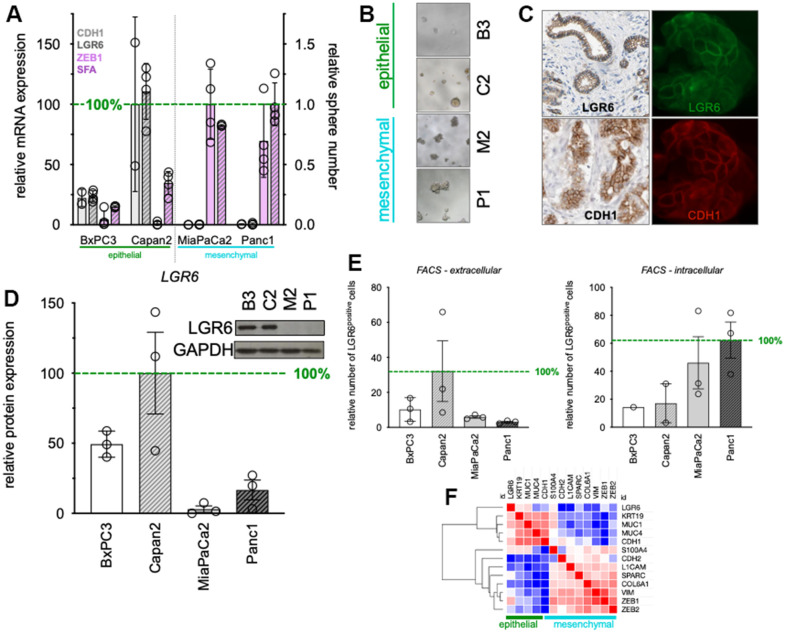
**Baseline expression of LGR6 in PDAC cell lines.** (**A**) qPCR of epithelial (*CDH1*), mesenchymal (*ZEB1*) markers, and *LGR6*. In addition, sphere-formation capacity (SFA) was evaluated. (**B**) Morphology of defined epithelial and mesenchymal PDAC cell lines; (**C**) Immunohistochemistry (left panels, Protein atlas) and immunofluorescence staining LGR6 (green) and E-cadherin (red) in Capan2; (**D**) Western blot analysis of LGR6 in pancreatic cancer cell lines. Quantification is shown as normalized to Capan2; (**E**) Flow cytometric analysis of extra- and intra-cellular LGR6 in pancreatic cancer cell lines; (**F**) Clustering analysis of the TCGA databank on PDAC identified *LGR6* with epithelial marker group *, P<0.05; **, P<0.01; ***, P<0.001; ****, P<0.0001.

PDAC cell lines were categorized into epithelial and mesenchymal phenotypes according to their morphology (Supplementary Figure 1) and expression patterns of the epithelial marker E-cadherin [33] or mesenchymal transcription factor ZEB1 [34]. According to this, BxPC3 and Capan2 phenotypically categorized as epithelial due to their cobblestone-like cell-cell connections and higher expression of *CDH1* (E-cadherin). In contrast, MiaPaCa2 and Panc1 showed lose cell-cell connectivity with spindle-shaped morphology, lower expression of *CDH1* (E-cadherin), high expression of *ZEB1* and high sphere-formation potential (SFA) classifying them as mesenchymal (Figure 1A, left). To understand LGR6 expression patterns in the above-mentioned categorized cell lines, *LGR6* gene expression was determined and sided with the epithelial markers (Figure 1A). As shown before, phenotypical differences were also detectable in 3D with epithelial spheres appearing very compact and mesenchymal spheres rather grape-like (Figure 1B) [32]. Immuno-fluorescence staining detected strong LGR6 signals on the cell membrane in epithelial cells (Figure 1C right panels) suggesting colocalization with E-Cadherin (CDH1) which is in line with immunohistochemical stains of public databases (Figure 1C left panels). Western blotting for LGR6 protein corroborated these findings and demonstrated up to 33-fold stronger expression (Capan2 vs. MiaPaCa2) in epithelial cells (Figure 1D).

In line with this, flowcytometric intra- (Figure 1E, left) and extra-cellular (Figure 1E, right) LGR6 expression patterns were analyzed. Epithelial subsets showed markedly higher membrane bound LGR6 (up to 10-fold, Capan2 vs. Panc1) and lower cytoplasmic LGR6 expression compared to the mesenchymal cell lines, where proportions were reversed (Figure 1E).

Last, database analysis of the TCGA databank on PDAC revealed strong hierarchical clustering of *LGR6* with epithelial markers *KRT19*, *MUC1*, *MUC4*, and *CDH1*, whereas mesenchymal markers robustly clustered separately (Figure 1F). GSEA analysis of typical EMT signatures corroborated these data (Supplementary Figure 2). Moreover, overlap analysis of TCGA and CCLE data, where we compared LGR6 high to LGR6 low expression, revealed 30 genes in common of both databases (Supplementary Figure 3). Several of those genes were related to cell-cell adhesion, such as cytokeratins or cell adhesion molecules (Supplementary Table 2).

### Heterogeneous WNT activity associates with LGR6 expression and epithelial signatures in PDAC

**Figure 2:**
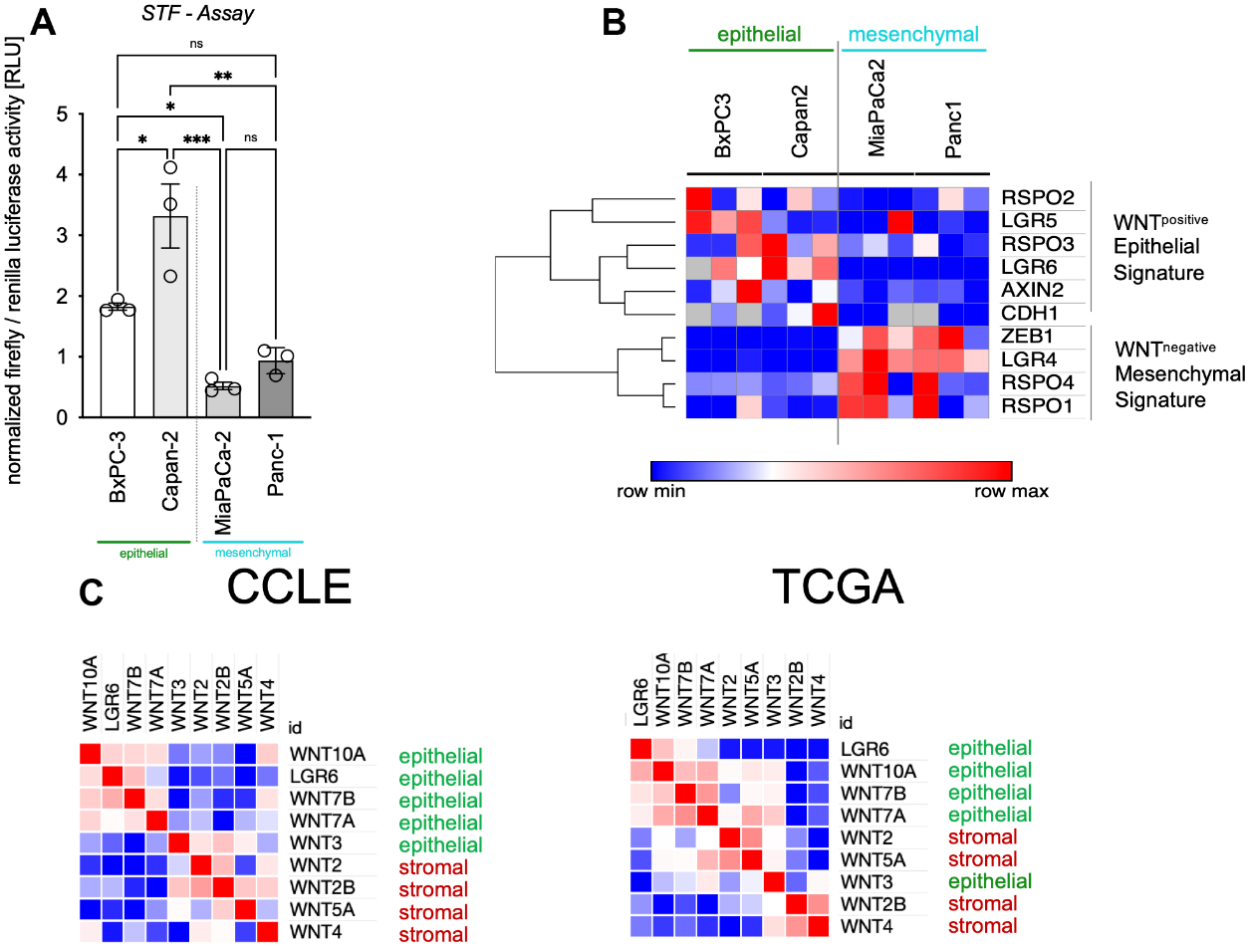
**LGR6 expression correlates with epithelial signatures.** (**A**) Super TOP/FOP assays (STF assay) revealed higher baseline WNT activity levels in epithelial (green) vs. mesenchymal (blue) pancreatic cancer cells. (**B**) Clustering of gene expression in the same cell lines affiliates *LGR6* with WNT^positive^ epithelial signature, whereas the WNT^negative^ mesenchymal signature was highly expressed in mesenchymal PDAC cell lines (blue). (**C**) Epithelial WNT niche subtypes described by Seino and colleagues clustered with *LGR6* in CCLE and TCGA datasets. *, P<0.05; **, P<0.01; ***, P<0.001.

As we could show before, WNT activity is very heterogenous in between different cell types of PDAC as well as different cells of the same cell line [32]. In this work, we show in Super TOP/FOP assays, that BxPC3 and Capan2 (epithelial category) tended to contain higher baseline WNT activity levels (Figure 2A). Clustering of mRNA results revealed an affiliation of *LGR6* with WNT target genes *AXIN2* and *LGR5* as well as *RSPO2*, *RSPO3*, and *CDH1* what we describe as WNT^positive^ epithelial signature. In contrast, *RSPO1*, *RSPO4*, *LGR4*, and *ZEB1* were highly expressed in mesenchymal cells as part of the WNT^negative^ mesenchymal signature (Figure 2B). According with this, we analyzed WNT niche subtypes described by Seino and colleagues in CCLE and TCGA datasets. Here, we also found that epithelial WNTs (3, 7A and B, and 10A) clustered strongly with *LGR6*. This might give hints towards a robust role of LGR6 creating WNT independence in those cell lines (Figure 2C) [35].

### Exogeneous canonical WNT activation mediates LGR6 expression

**Figure 3:**
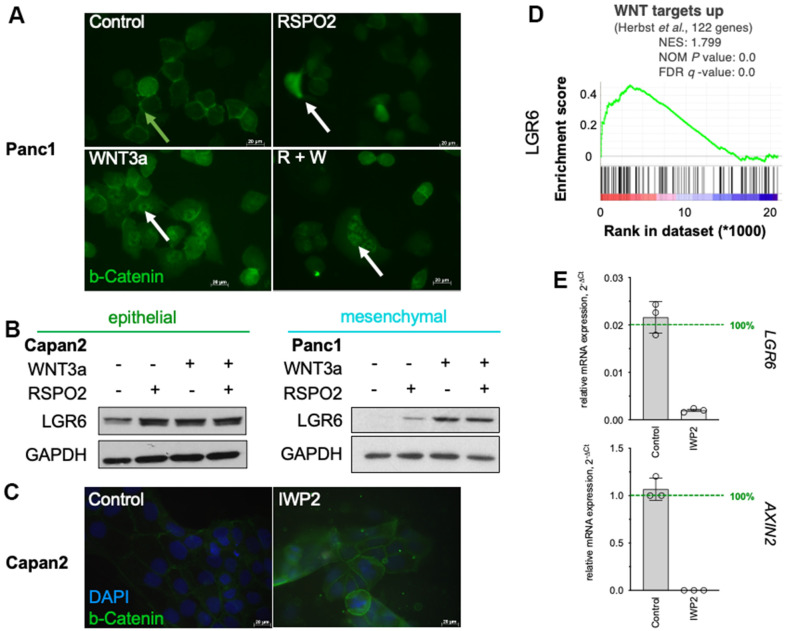
**LGR6 as a WNT target gene.** (**A**) Immunofluorescence staining of β-catenin (green) in Panc1 after stimulation with RSPO2, WNT3a or its combination (R+W) showed more nuclear staining upon WNT activation, in particular with WNT3a or R+W. White arrows indicate nuclear β-catenin, green arrows indicate membrane-bound β-catenin. (**B**) Western blot of LGR6 after similar stimulation showed increase in both Capan2 and Panc1. (**C**) GSEA of TCGA data revealed positive correlation of *LGR6* with the WNT signature “WNT targets up” by Herbst et al. (**D**) Immunofluorescence staining of β-catenin in Capan2 reveals increase in membrane-bound and decrease of nuclear β-catenin upon IWP2 inhibition. The cells were cultured with 10uM IWP2 for 72h, medium was changed every other day. Bar, 20 μm; (**E**) Effect of IWP2 on *AXIN2* and *LGR6* mRNA expression in Capan2. Significant decreases were detected in presence of IWP2. ***, P<0.001; ****, P<0.0001.

As described before, exogenous WNT3a and RSPO2 enhance WNT signaling in highly responsive cells, whereas RSPO2 alone seems insufficient in most cell lines [32]. Immunofluorescent assays confirmed this notion and showed beta-Catenin translocation into the nucleus after co-stimulation in Panc1 (Figure 3A). Western blotting for LGR6 demonstrated upregulation upon exogeneous WNT stimulation with stronger effects with both RSPO2 and WNT3a (Figure 3B). Gene Set Enrichment Analysis (GSEA) also revealed a strong positive correlation of WNT targets and LGR6 in PDAC supporting our hypothesis that *LGR6* might be a novel WNT target gene in PDAC (Figure 3C). To further investigate the regulatory effects of WNT signaling on LGR6 expression, we reduced WNT signaling activity using the porcupine inhibitor IWP2. IWP-2 selectively targets porcupine, a membrane-bound acyltransferase (MBOAT), which is essential to produce WNT proteins. Administration of IWP-2 leads to inhibition of LRP6 and Dvl2 phosphorylation and decreased beta-Catenin accumulation [32]. These effects are transient and reversible [36].

As shown in Figure 3D, we detected decreased β-catenin signal upon IWP2 inhibition in Capan2. Moreover, down-regulation of the WNT target gene *AXIN2* in Capan2 corroborated this finding on RNA level (Figure 3E). A reduced *LGR6* expression was detected upon the inhibition of WNT activity (Figure 3E). These data suggest that downregulation of WNT signaling might be followed by a reduction of LGR6 expression.

### LGR6 knock-down reduces cancer stemness and responsiveness to exogenous WNT stimulation in PDAC

**Figure 4:**
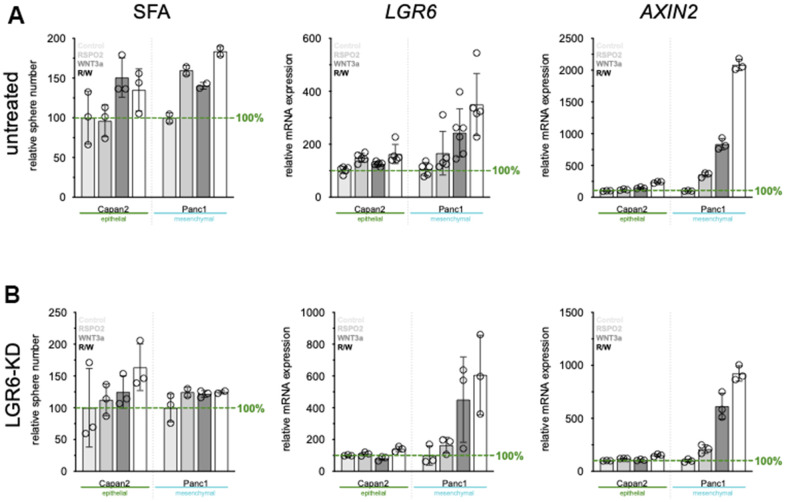
**LGR6 depletion correlates with reduced cancer stemness.** (**A**) sphere formation ability (SFA) and qPCR of *LGR6* and WNT target gene *AXIN2* in vector control treated cells. Stimulation with RSPO2, WNT3a or its combination leads to increased SFA, and *LGR6/AXIN2*. Knock-down of *LGR6* (**B**) reduces SFA and influences *LGR6* and *AXIN2* gene expression, suggesting partial regulation of canonical WNT through LGR6.

To fully explore functional roles of LGR6 in cancer cell stemness, LGR6 was knocked down by siRNA transfection. Efficient knock down resulted in a 90% decrease in *LGR6* mRNA level in comparison to the vector control (Supplementary Figure 4A). Next, we evaluated the effect of LGR6 silencing in PDAC cell lines on their colony formation ability. As shown in Supplementary Figure 4B, colonies were markedly smaller in the siLGR6-transfected group (siLGR6) compared to the vector control (VC). Consistent with this result, SFAs revealed that siLGR6 led to a decrease in spheroid size and number compared with VC (Supplementary Figure 4C). To investigate the role of LGR6 in exogeneous mediation of WNT, we also evaluated SFA, *LGR6*, and WNT target *AXIN2* expression after stimulation with RSPO2 and WNT3a after VC (Figure 4A) or LGR6-KD (Figure 4B) in both Capan2 and Panc1. As expected, SFA, *LGR6*, and the WNT target gene *AXIN2* significantly increased upon RSPO2 and/or WNT3a stimulation, indicating a successful WNT axis activation (Figure 4A). LGR6-KD impeded sphere-formation even after exogeneous WNT stimulation, especially in the more WNT-sensitive cell line Panc1 (Figure 4B). *LGR6* and *AXIN2* expression were lower, also after exogeneous WNT stimulation; however, both were not completely inhibited suggesting an important, but not all-encompassing role of LGR6 in regulating the canonical WNT axis in PDAC (Figure 4B).

### LGR6 mRNA expression is associated with malignancy in PDAC and a trend towards worse survival

**Figure 5:**
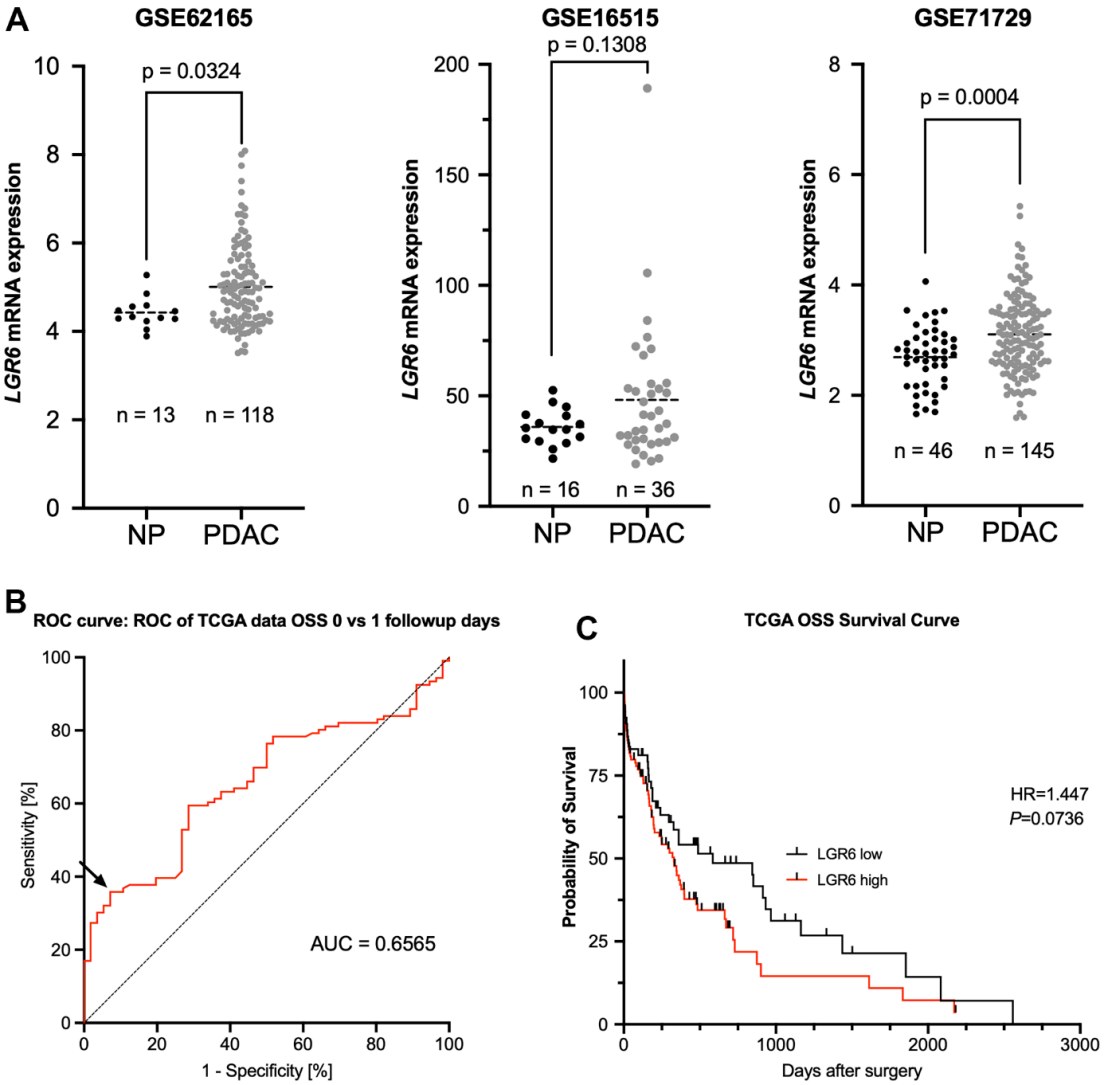
**LGR6 is higher expressed in PDAC compared to normal tissue and trends towards worse overall survival.** (**A**) Analysis of publicly available datasets (GSE62165, GSE16515, and GSE71729) shows significantly more *LGR6* in tumor vs. normal pancreatic tissue (NP). (**B**) ROC curve analysis of normalized *LGR6* mRNA expression for best discrimination threshold with an area under the curve (AUC) of 0.6565. (**C**) Dichotomal classification revealed a trend of high LGR6 expression and poor overall survival; survival of PDAC patients of LGR6^high^ versus LGR6^low^ is decreased without statistical significance.

Finally, we tested for clinical correlations of *LGR6* mRNA levels in different publicly available data sets with a total of 299 PDAC and 75 normal pancreas (NP) cases. *LGR6* expression was significantly higher in PDAC as compared to NP in GSE62165 and GSE71729 with a similar trend in GSE16515 (Figure 5A). Using ROC curve analyses and Youden’s index, we identified an ideal cutoff at a normalized expression intensity of 0.6565 (natural scale) of *LGR6* mRNA (Figure 5B). Dichotomal classification revealed a trend of high *LGR6* expression and poor overall survival (Figure 5C). Expressed in numbers, median survival of the LGR6 high subgroup was 329 days, whereas LGR6 low led to an increased survival with a median of 584 days. However, the remaining clinical data of the TCGA databank revealed no statistically significant difference (Supplementary Table 3).

## DISCUSSION

The canonical WNT pathway is reportedly an essential protagonist in organ development as well as oncogenesis in multiple cancers. LGR5 was initially identified as a WNT target gene in human colon cancer and especially in those that harbored WNT-activating mutations [37]. Moreover, it has been shown to act as a receptor of RSPO and as such, LGR5 enhances the canonical WNT pathway [29, 31]. The RSPOs are a family of secreted factors that augment already activated WNT signaling through binding to LGR4-6. Our previous study indicated that RSPO2 could enhance WNT activity of certain PDAC cells and endow highly responsive subgroups with CSCs characteristics [32]. As a close homolog of LGR5, we hypothesized that LGR6 might be an essential requirement in PDAC for reasons other than its assumed role as a receptor of RSPOs and enhancer of WNT signaling. In the present study, we were able to demonstrate that LGR6 expression was altered with differential WNT activity. This led to the idea that the *LGR6* gene itself may be a direct target of the WNT signaling pathway in PDAC cells as we were able to show that activated WNT led to enhanced LGR6 expression; vice versa, WNT inhibition was associated with decreased expression patterns of LGR6. LGR6 regulation in PDAC may therefore constitute a crucial role within a positive feedback loop of WNT signaling activity.

Previous research showed that overexpression or knockout of LGR5 resulted in pronounced changes of the cytoskeleton and cell adhesion complexes in some cancer cell lines lacking endogenous or exogenous RSPO stimulation [38, 39]. LGR5 silenced colorectal cancer cells tended to be more mesenchymal, while overexpression of LGR5 was linked to a more epithelial phenotype [38]. LGR5 overexpression in hepatocellular carcinoma cells resulted in changes from a mesenchymal phenotype to a more aggregated phenotype typical for the epithelial subtype. Knockdown of LGR5 shifted cells from an aggregated phenotype to a spindle-shaped one [39]. On the other hand, overexpression of LGR6 in HeLa cells increased cell movement after treatment with RSPO1 and WNT3a. Moreover, overactivation of WNT signaling correlates with increased cell migration [40]. To investigate these – in part - conflicting findings, we submerged into more detail in the present study. We used PDAC cell lines of different morphological appearances to study the association of LGR6 in EMT subgroups of PDAC. In this regard, Capan2 and BxPC3 represent an epithelial phenotype with intensive cell-cell contacts, while MiaPaCa2 and Panc1 exemplify the mesenchymal phenotype with spindle-shaped morphology and lose cell-cell contacts [32, 41]. Using multiple approaches, we found that LGR6 is preferentially expressed in more epithelial cell lines, whereas mesenchymal cell lines harbored much lower LGR6 expression levels. Furthermore, expression pattern analysis of LGR6 showed that its distribution was mostly located on the cell membrane in epithelial cell lines, while less or no expression was detected in mesenchymal cell lines in this position.

Cancer stem cells (CSCs) are believed to initiate and maintain malignancies of different types and engage in chemo-resistance mechanisms as well as metastatic activities. LGR5 was identified as an adult stem cell marker in various tissues, including intestine, liver, skin, stomach, and ovarian epithelia [42, 43]. Both LGR5+ and LGR6+ stem cell compartments contribute to epidermal repair in response to acute wounds [44, 45]. In the present study, we investigated stemness PDAC cell lines by typical *in vitro* assays, such as colony formation capacity in 2D or sphere formation capacity in 3D. The deletion of LGR6 negatively impacted on the growth of colonies and spheres, indicating that LGR6 might have a potential role in maintaining PDAC stemness. In part, this could also be explained by reduced canonical WNT activity after LGR6 knock down.

There are several limitations to the present study. First, we did not generate any LGR6-overexpressing PDAC cell lines for further functional analysis. Second, comparison of LGR6+ with LGR6- cells after flowcytometric cell sorting might be another elegant way to explore the roles of LGR6 in WNT signaling, EMT, and cancer stemness. Third, mouse models have not been implemented to translate our *in vitro* results into *in vivo* conditions where essential mechanisms, such as metastasis or chemo-resistance could be further evaluated. In a clinical translational approach, we believe that correlating gene or protein expression with different genomic as well as metabolic subtypes [46–48] could further enlighten the role of LGR6. Stratification of PDAC after primary resection into LGR6 ^high^ versus LGR6 ^low^ tumors and/or organoids could further help to decide whether enforced chemotherapeutic regimens such as FOLFIRINOX should be applied.

Taken together, we present new evidence in PDAC that LGR6 might be a novel WNT target gene in this tumor. LGR6 seems to be involved in EMT and cancer stemness. This knowledge could be applicable for detection and treatment of special subsets of pancreatic cancer cells. Further research is still needed to dissect the exact mechanisms under physiological as well as pathological conditions of benign and cancerous pancreatic cells.

## Supplementary Material

Supplementary Figures

Supplementary Tables
