# Supplementary material for: WNT enhancing signals in pancreatic cancer are transmitted by LGR6
Source: Aging (Albany NY). 2023 Sep 27;15(20):10897–914. doi: 10.18632/aging.205101 (PMC10637827; doi:10.18632/aging.205101)
Supplement: Supplementary Figures [file aging-15-205101-s001.pdf]

## SUPPLEMENTARY FIGURES

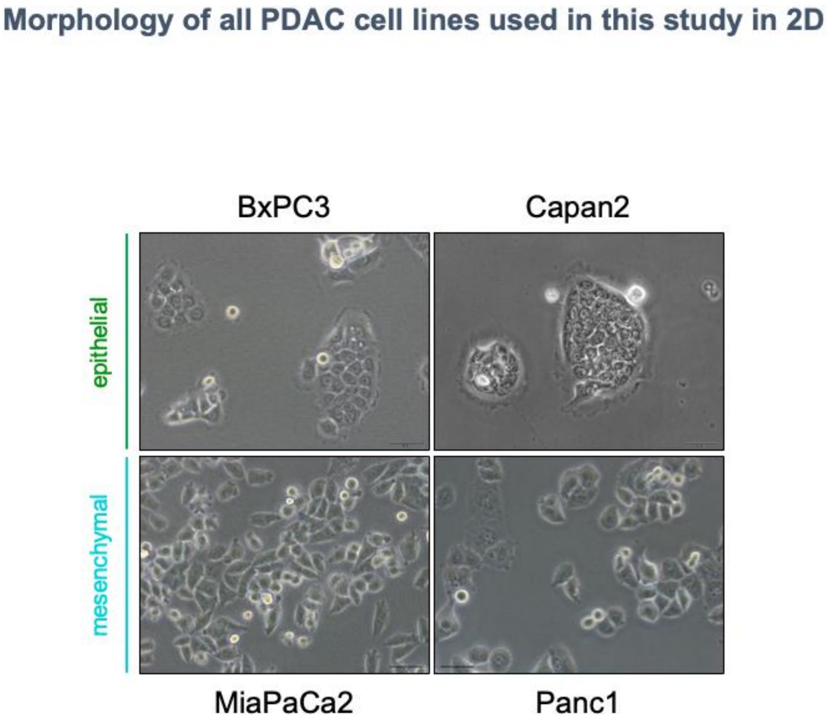


**Supplementary Figure 1. 2D-Morphology of PDAC cell lines used in this study.** Grouping according to epithelial (green; BxPC3, Capan2) or mesenchymal (blue; MiaPaCa2, Panc1) phenotype.

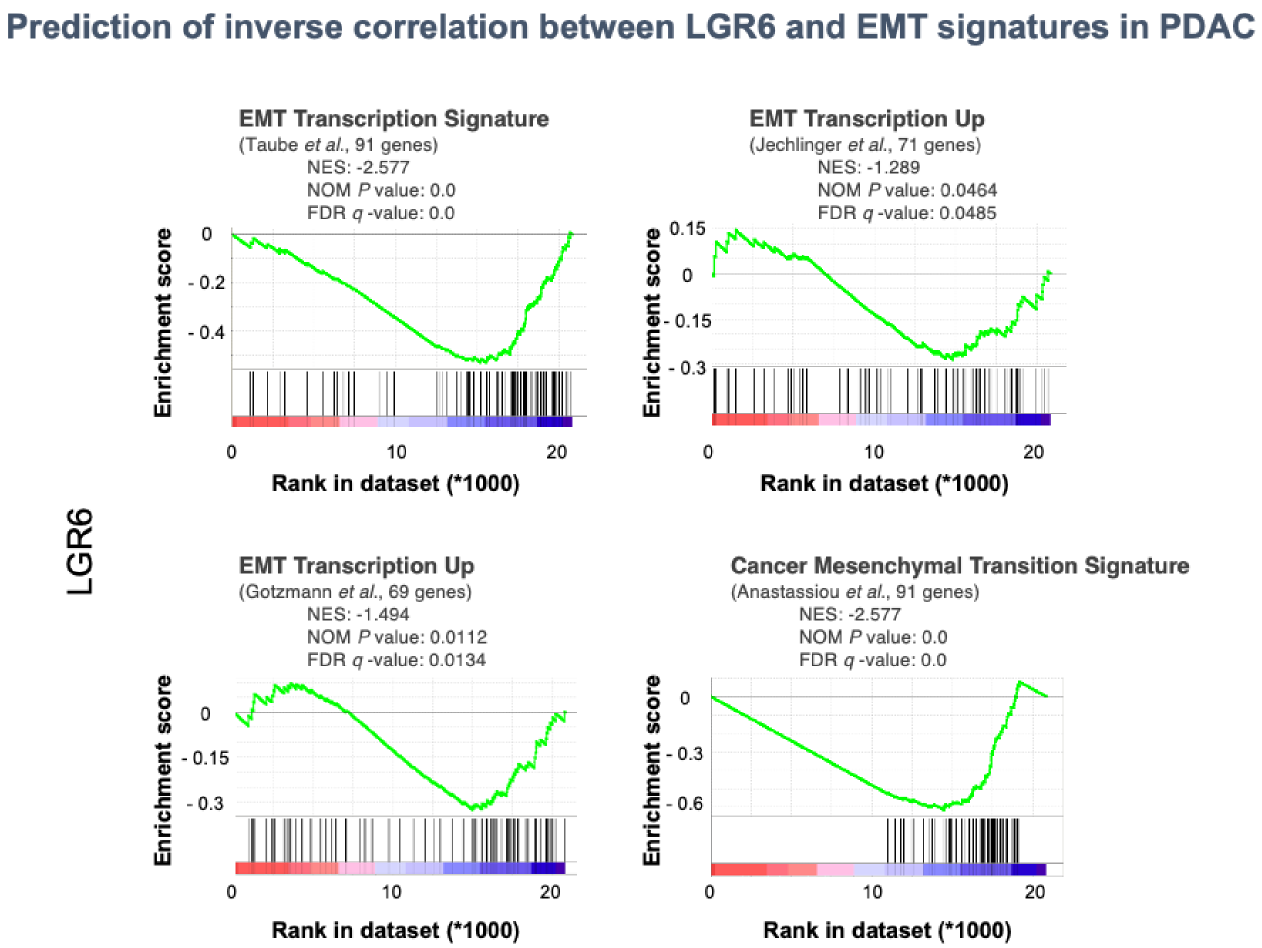


**Supplementary Figure 2. GSEA analysis of typical EMT signatures in TCGA data (PDAC).**

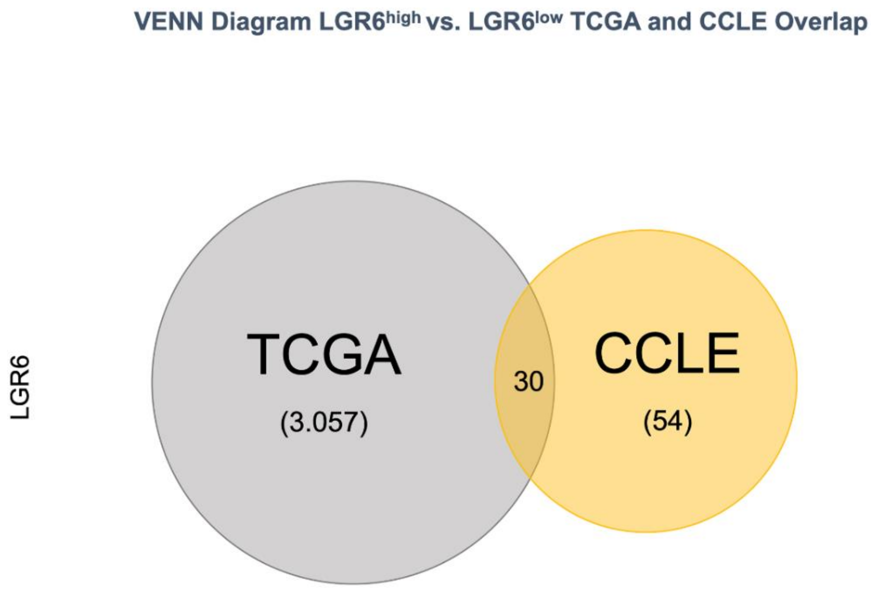


**Supplementary Figure 3. VENN diagram of overlap analysis (TCGA in grey and CCLE in yellow); comparison of LGR6high to LGR6low expression (overlapping genes are listed in Supplementary Table 2).**

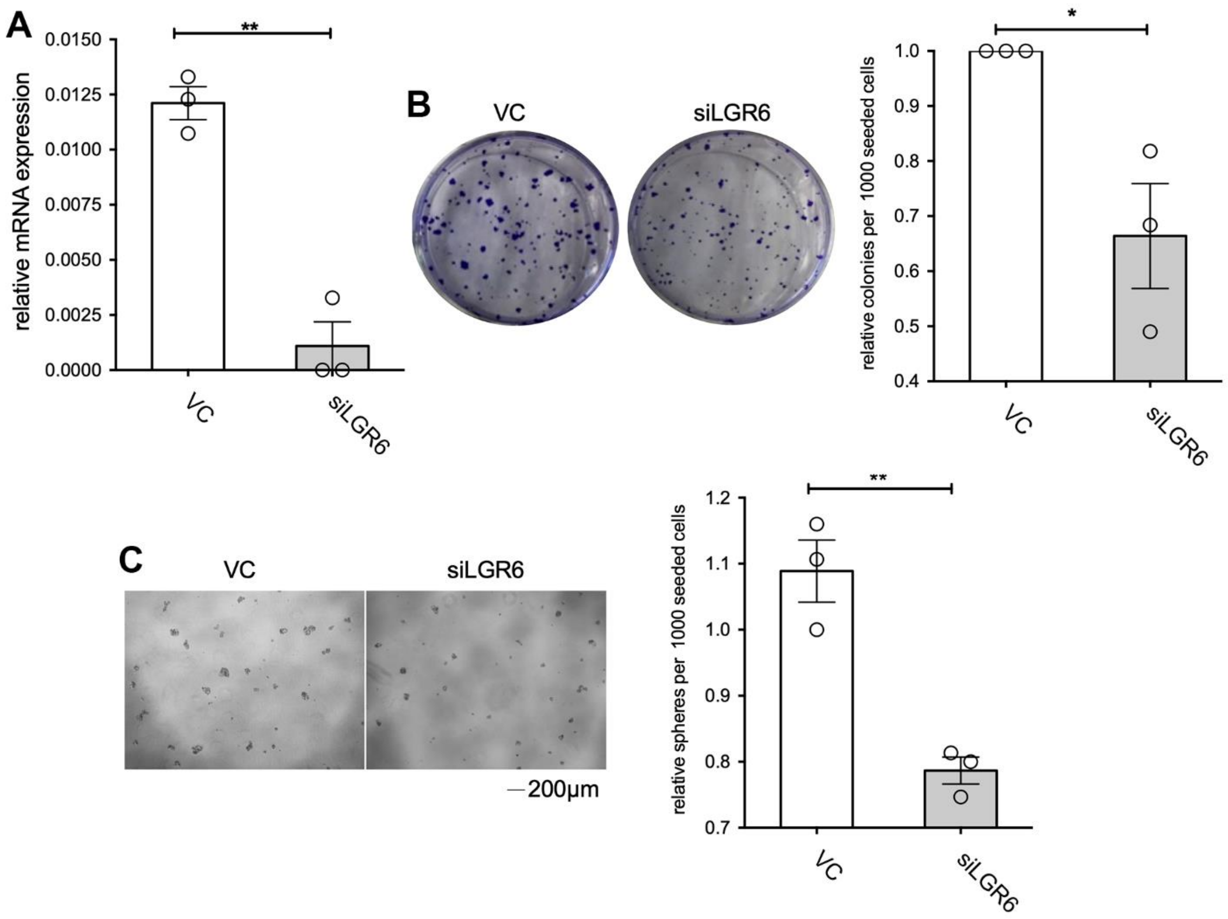


**Supplementary Figure 4. LGR6 knock-down reduces features of cancer stemness in PDAC.** (**A**) Knock-down (KD) efficiency was tested by PCR for LGR6 mRNA levels; vector control (VC) vs. siLGR6 revealed significant KD. (**B**) colony-formation units and (**C**) sphere-formation ability after VC or siLGR6 treatment was carried out and resulted in decreased and smaller colony or sphere numbers.
