# Supplementary material for: WNT enhancing signals in pancreatic cancer are transmitted by LGR6
Source: Aging (Albany NY). 2023 Sep 27;15(20):10897–914. doi: 10.18632/aging.205101 (PMC10637827; doi:10.18632/aging.205101)
Supplement: Supplementary Tables [file aging-15-205101-s002.pdf]

## SUPPLEMENTARY TABLES

**Supplementary Table 1. Antibodies used in this study.**

| Antibodies | Brand | Application | Dilution |
|---|---|---|---|
| LGR6 | Sigma, HPA008556 | WB | 1:1000 |
| E-Cadherin | BD Biosciences, 610181 | WB | 1:1000 |
| beta-Catenin | Cell Signaling Technologies (CST) #8480 | WB | 1:1000 |
| phospho-beta-Catenin (Ser675) | CST #4176 | WB | 1:1000 |
| GAPDH | Santa Cruz Biotechnology (SCBT), sc-25778 | WB | 1:1000 |
| Mouse IgG-HRP | SCBT, sc-2005 | WB | 1:2500 |
| Rabbit IgG-HRP | CST, #7074 | WB | 1:2500 |
| LGR6 | Sigma, HPA008556 | IF | 1:50 |
| beta-Catenin | CST, #8480 | IF | 1:100 |
| Mouse IgG-FITC | SCBT, sc-2010 | IF | 1:100 |
| Rabbit IgG-FITC | SCBT, sc-2012 | IF | 1:100 |
| Mouse IgG-TR | SCBT, sc-2781 | IF | 1:100 |
| LGR6 APC-conjugated | R&D Systems, FAB8458A-025 | Flowcytometry | Acc. to manufacturer |

**Supplementary Table 2. Common genes of overlap analysis (TCGA and CCLE data) with comparison of LGR6high to LGR6low expression (related to VENN diagram in Supplementary Figure 3).**

| ID | Full name |
|---|---|
| AKR1B10 | aldo-keto reductase family 1 member B10 |
| ALDH1A1 | aldehyde dehydrogenase 1 family member A1 |
| ALDH3A1 | aldehyde dehydrogenase 3 family member A1 |
| ANO1 | anoctamin 1 |
| BCHE | butyrylcholinesterase |
| BMP4 | bone morphogenetic protein 4 |
| BST2 | bone marrow stromal cell antigen 2 |
| CEACAM5 | CEA cell adhesion molecule 5 |
| CEACAM6 | CEA cell adhesion molecule 6 |
| CLIC3 | chloride intracellular channel 3 |
| CXCL14 | C-X-C motif chemokine ligand 14 |
| DPP4 | dipeptidyl peptidase 4 |
| EDAR | ectodysplasin A receptor |
| FGF19 | fibroblast growth factor 19 |
| FGFBP1 | fibroblast growth factor binding protein 1 |
| GPX3 | glutathione peroxidase 3 |
| KLK10 | kallikrein related peptidase 10 |
| KRT14 | keratin 14 |
| KRT17 | keratin 17 |
| KRT6A | keratin 6A |
| LY6D | lymphocyte antigen 6 family member D |
| MSLN | mesothelin |
| PLA2G10 | phospholipase A2 group X |
| PRR15 | proline rich 15 |
| PSCA | prostate stem cell antigen |
| S100P | S100 calcium binding protein P |

| | |
|---|---|
| SERPINB5 | serpin family B member 5 |
| SPESP1 | sperm equatorial segment protein 1 |
| SPRR1B | small proline rich protein 1B |
| SPRR3 | small proline rich protein 3 |

**Supplementary Table 3. Clinical data (TCGA) in correlation with LGR6 mRNA expression in pancreatic ductal adenocarcinoma.**

| Characteristics | Total | *LGR6* Low | *LGR6* High | *P* |
|---|---|---|---|---|
| All patients | 178 (100.0) | 62 (34.8) | 116 (65.2) | |
| **Gender** | | | | |
| Male | 95 (56.5) | 33 (34.7) | 62 (65.3) | 0.87 |
| Female | 73 (43.5) | 24 (32.9) | 49 (67.1) | |
| **T-category** | | | | |
| T1 | 8 (5.0) | 1 (12.5) | 7 (87.5) | 0.5696 |
| T2 | 19 (11.8) | 12 (63.2) | 7 (36.8) | |
| T3 | 131 (81.4) | 42 (32.1) | 89 (67.9) | |
| T4 | 3 (1.8) | 1 (33.3) | 2 (66.7) | |
| **Nodal Metastasis** | | | | |
| Negative | 42 (26.6) | 15 (35.7) | 27 (64.3) | 0.8504 |
| Positive | 116 (73.4) | 39 (33.6) | 77 (66.4) | |
| **Distant Metastasis** | | | | |
| Negative | 74 (45.7) | 26 (35.1) | 48 (64.9) | >0.9999 |
| Postitive | 88 (54.3) | 31 (35.2) | 57 (64.8) | |
| **Diabetes** | | | | |
| Negative | 98 (73.1) | 35 (35.7) | 63 (64.3) | >0.9999 |
| Positive | 36 (26.9) | 13 (36.1) | 23 (63.9) | |
| **Alcohol history** | | | | |
| Negative | 57 (37.5) | 22 (38.6) | 35 (61.4) | 0.7277 |
| Positive | 95 (62.5) | 33 (34.7) | 62 (65.3) | |
| **History Chronic Pancreatitis** | | | | |
| Negative | 117 (90.7) | 43 (36.8) | 74 (63.2) | >0.9999 |
| Positive | 12 (9.3) | 4 (33.3) | 8 (66.7) | |

Values in parentheses indicate column and row percentage for total and *LGR6* low or high cases, respectively.
